# Liver metastasis from follicular thyroid carcinoma successfully treated with hepatectomy and tyrosine kinase inhibitor: a case report

**DOI:** 10.1093/jscr/rjag387

**Published:** 2026-05-27

**Authors:** Hian Ee Heng, Tak Kuan Chow, Khoon Leong Ng, Peng Soon Koh

**Affiliations:** Department of Surgery, Faculty of Medicine, University Malaya, Lembah Pantai, 50603, Kuala Lumpur, Malaysia; Department of Pathology, Faculty of Medicine, University Malaya, Lembah Pantai, 50603, Kuala Lumpur, Malaysia; Department of Surgery, Faculty of Medicine, University Malaya, Lembah Pantai, 50603, Kuala Lumpur, Malaysia; Department of Surgery, Faculty of Medicine, University Malaya, Lembah Pantai, 50603, Kuala Lumpur, Malaysia

**Keywords:** metastatic follicular thyroid carcinoma, liver metastasis, liver metastasectomy, tyrosine kinase inhibitor

## Abstract

Liver metastasis from differentiated thyroid cancer (DTC) is rare, occurring in about 0.5% of metastatic cases. We hereby report a 32-year-old woman with widely invasive follicular thyroid carcinoma (FTC) who underwent total thyroidectomy followed by adjuvant radioactive iodine therapy. Two years later, she developed multiple neck recurrences requiring repeat surgeries, complex reconstruction, and adjuvant radiotherapy. Despite complete resection, her serum thyroglobulin (SrTg) remained elevated. Radioiodine whole-body scan (WBS) and fluorodeoxyglucose - positron emission tomography (FDG-PET) imaging revealed liver metastasis. She subsequently underwent elective left hemi-hepatectomy, and histology confirmed metastatic FTC. Postoperatively, lenvatinib therapy was initiated. Three years after surgery, she remains well, with no recurrence on FDG-PET and declining SrTg levels. This case highlights the value of WBS and FDG-PET in detecting occult metastases in patients with persistent SrTg elevation. It also demonstrates that combining liver metastasectomy with tyrosine kinase inhibitor therapy (lenvatinib) may improve outcomes in selected patients with DTC and liver metastasis.

## Introduction

Thyroid cancer is the most common endocrine malignancy, with rising incidence over the past four decades [1]. Differentiated thyroid cancer (DTC), comprising papillary thyroid carcinoma (PTC) and follicular thyroid carcinoma (FTC), accounts for over 85% of cases [2]. FTC is the second most common subtype (12%) and typically spreads hematogenously, with distant metastases reported in 7%–23% of cases, most commonly to the lungs and bones [3]. Distant metastasis may occur at presentation or during disease progression and is the most significant prognostic factor [4, 5].

Liver metastasis from FTC usually appears in advanced disease with widespread metastases and is often associated with radioiodine-refractory disease and poor prognosis [6]. Isolated liver metastasis, however, is rare, accounting for ~0.5% of all metastatic cases. Previous reports of PTC with liver metastasis described late recurrences many years after thyroidectomy, whereas our patient developed FTC liver metastasis within 3 years, likely reflecting the hematogenous spread typical of FTC compared with the lymphatic spread seen in PTC [7, 8]. Surgical metastasectomy, when feasible, combined with tyrosine kinase inhibitor (TKI) therapy targeting vascular endothelial growth factor (VEGF) pathways may help to achieve disease control and improve survival [9]. We report a case of widely invasive FTC with isolated liver metastasis treated successfully with liver resection followed by lenvatinib therapy.

## Case presentation

A 32-year-old Chinese woman with no family history of malignancy was diagnosed with widely invasive FTC. She underwent total thyroidectomy, and histology showed a solitary tumor measuring 6.0 × 3.0 × 4.3 cm with an intact capsule (T3N0M0, stage I). She received adjuvant radioiodine ablation and achieved remission, with serum thyroglobulin (SrTg) 2.9 ng/mL and a negative radioiodine whole-body scan (WBS).

Two years later, she developed a neck recurrence with SrTg rising to 3000 ng/mL. She underwent wide local excision with right deep circumflex iliac myocutaneous free flap reconstruction. Histology confirmed recurrent FTC with a 1 mm margin, followed by adjuvant radiotherapy (63 Gy in 28 fractions). After being lost to follow-up for 1 year, she returned with SrTg >30 000 ng/mL. Imaging revealed persistent neck disease, and a third surgery achieved 1 cm margin clearance.

Despite adequate resection, she remained in persistent disease with SrTg remained elevated (>30 000 ng/mL). Fluorodeoxyglucose - positron emission tomography (FDG-PET) and radioiodine WBS (Figs 1–3) showed no neck disease but detected a mixed iodine- and FDG-avid metastasis in the left liver lobe. Following multidisciplinary team discussion, in view of patient’s young age with aggressive radioiodine refractory disease as she had radioactive iodine after the first surgery and still develop tumor recurrence with metastatic disease, she was subjected for an open left hemi-hepatectomy. The tumor was confined to segments II–III with no other intra-abdominal metastases (Figs 4 and 5). Histology confirmed metastatic FTC measuring 12.0 × 10.5 × 6.0 cm with 1 mm margin clearance (Fig. 6).

**Figure 1:**
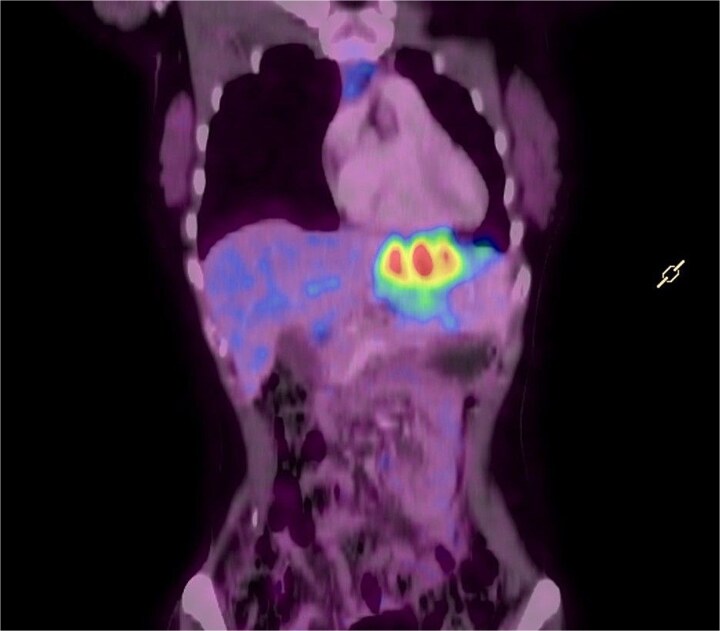
Showed an image of an axial view of PET-CT with hypermetabolic enhancing left liver lobe mass (SUV_max_ 9.1/7.1 × 10.9 × 8.5 cm) indicating recurrent follicular thyroid carcinoma with left liver lobe metastasis.

**Figure 2:**
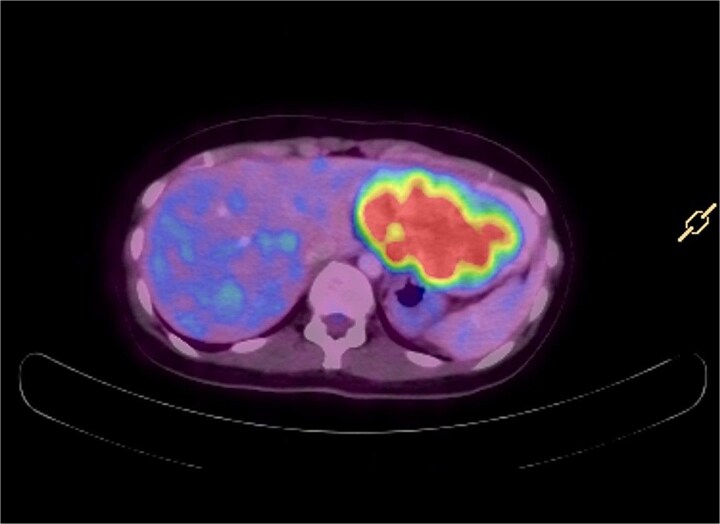
Showed an image of a axial view of PET-CT with hypermetabolic enhancing left liver lobe mass (SUV_max_ 9.1/7.1 × 10.9 × 8.5 cm) indicating recurrent follicular thyroid carcinoma with left liver lobe metastasis.

**Figure 3:**
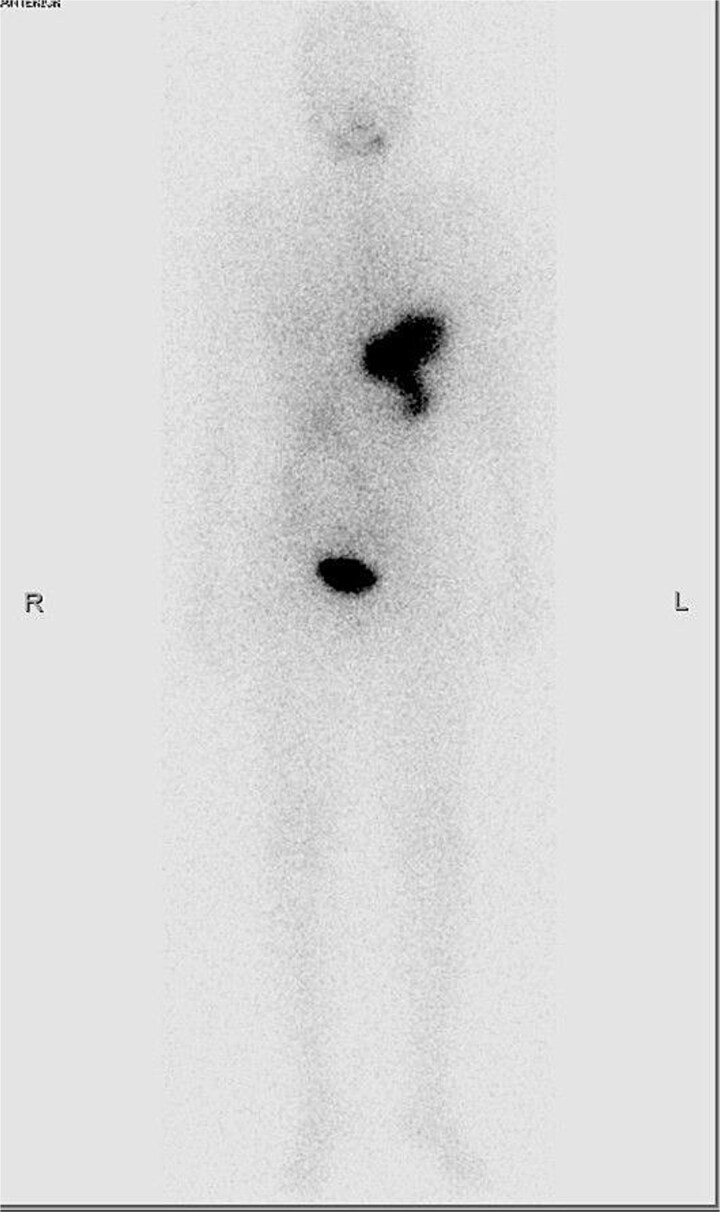
Shows an intense area of radioactive iodine uptake at the left upper quadrant on whole body planar scan suggestive of abdominal metastasis of follicular thyroid metastasis.

**Figure 4:**
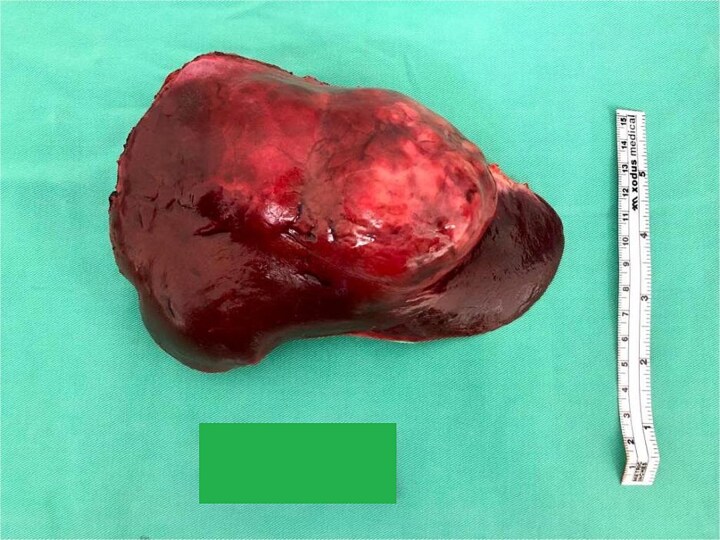
Showed the anterior view of the specimen of left hemi-hepatectomy with an obvious tumor occupying almost the entire left lobe of liver.

**Figure 5:**
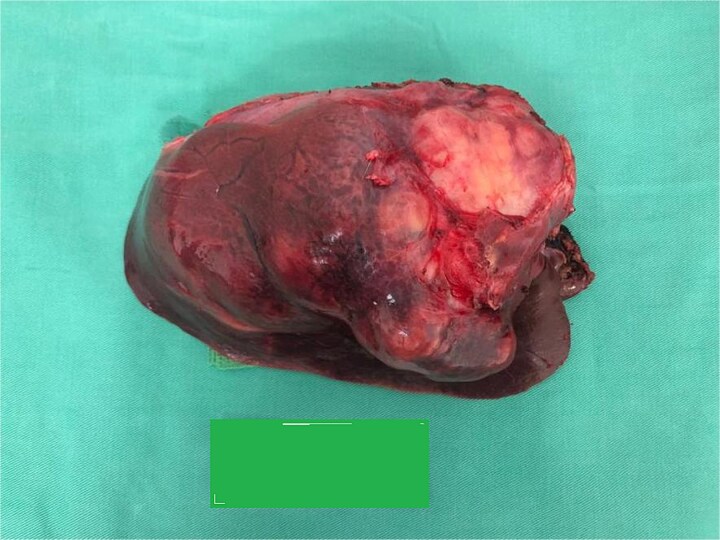
Showed the posterior view of the specimen of left hemi-hepatectomy with an obvious tumor occupying almost the entire left lobe of liver.

**Figure 6:**
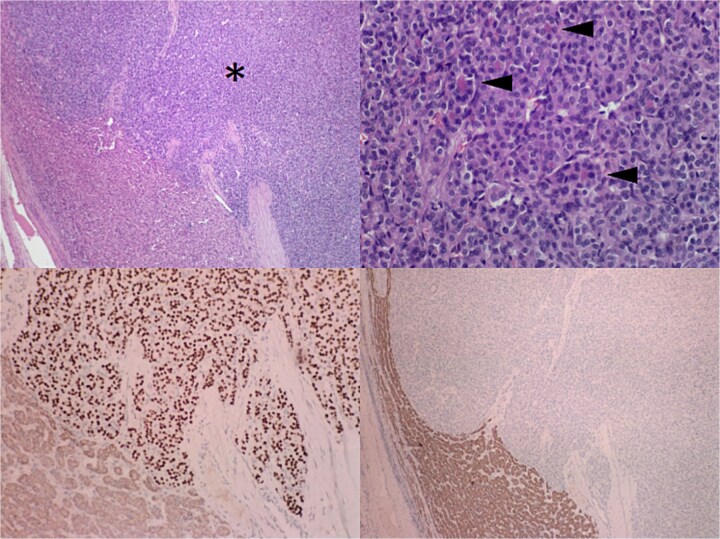
Showed the malignant follicular cells invading the liver parenchyma with was further confirmed with diffuse staining for TTF-1 and negative for hepatocyte specific antigen (HSA) which rules out hepatocellular carcinoma. (top left) histology of the excised liver segment showing malignant follicular cells (*) infiltrating the liver parenchyma (H&E, 40×). (Top right) the malignant cells form solid and trabecular growth patterns with occasional follicular structures. The arrows showing cell nuclei that are rounded and hyperchromatic, lacking nuclear longitudinal grooves and pseudoinclusions (H&E, 200×). (Bottom left) the tumor cells show strong and diffuse nuclear staining pattern with thyroid transcription factor 1 (TTF-1, 100×). (Bottom right) the tumor cells are however negative for hepatocyte specific antigen (HAS) (HAS 40×).

She was started on lenvatinib 10 mg daily and levothyroxine 100 mcg for thyroid stimulating hormone suppression. At 36-month follow-up, she remains well with no recurrence on FDG-PET. Her SrTg declined from >30 000 ng/mL to 55 ng/mL with TgAb <20 IU/mL, indicating biochemical and radiological remission.

## Discussion

Liver metastasis in differentiated thyroid cancer (LMDTC) is rare, resulting in limited management guidelines. In the literature, FTC with liver metastasis usually represents advanced disease with poor prognosis. Brient *et al.* reported a case series of 14 patients with LMDTC, in which 50% originated from FTC and only one patient underwent chemotherapy followed by liver resection, surviving 18 months postoperatively before dying from pneumonia [10].

Diagnosis of LMDTC is typically suggested by persistently elevated SrTg together with imaging such as radioiodine WBS, FDG-PET, or CT [11]. Radioiodine WBS is useful only for iodine-avid lesions that may respond to radioiodine therapy, whereas dedifferentiated tumors often lose iodine avidity; in such cases, FDG-PET is recommended to detect metastases [11, 12]. In our patient, persistent SrTg elevation prompted imaging, and liver metastasis was identified on both FDG-PET and radioiodine WBS. Histology confirmed metastatic FTC with malignant follicular cells and positive thyroid transcription factor-1 (TTF-1) staining, with clear surgical margins.

One month after surgery, the patient was started on the TKI lenvatinib. TKIs act as anti-angiogenic agents by targeting VEGF, thereby inhibiting tumor angiogenesis and hematogenous spread in FTC. Brient *et al.* demonstrated improved overall survival in patients with LMFTC treated with TKI therapy (28 months vs. 4 months without TKI) [10].

## Conclusions

Liver metastasis in FTC is rare. However, persistent elevation of SrTg should prompt thorough evaluation to identify recurrent or metastatic disease. In cases of non–radioiodine-avid metastases, FDG PET-CT is useful for disease localization. Although metastatic FTC generally indicates poor prognosis, surgical resection combined with TKI therapy may improve survival outcomes, as demonstrated in our case.
